# Comparison of Attenuated Total Reflectance Mid-Infrared, Near Infrared, and ^1^H-Nuclear Magnetic Resonance Spectroscopies for the Determination of Coffee's Geographical Origin

**DOI:** 10.1155/2017/7210463

**Published:** 2017-11-01

**Authors:** Jessica Medina, Diana Caro Rodríguez, Victoria A. Arana, Andrés Bernal, Pierre Esseiva, Julien Wist

**Affiliations:** ^1^Chemistry Department, Universidad del Valle, AA 25360, Cali, Colombia; ^2^Institut de Police Scientifique, Ecole des Sciences Criminelles, Université de Lausanne, 1015 Lausanne, Switzerland; ^3^Grupo de Investigación Ciencias, Educación y Tecnología-CETIC, Programa de Química, Facultad de Ciencias Básicas, Universidad del Atlántico, Km 7 Antigua Vía Puerto Colombia, Barranquilla, Atlántico, Colombia; ^4^Departamento de Ciencias Básicas, Universidad Jorge Tadeo Lozano, Bogotá, Colombia

## Abstract

The sensorial properties of Colombian coffee are renowned worldwide, which is reflected in its market value. This raises the threat of fraud by adulteration using coffee grains from other countries, thus creating a demand for robust and cost-effective methods for the determination of geographical origin of coffee samples. Spectroscopic techniques such as Nuclear Magnetic Resonance (NMR), near infrared (NIR), and mid-infrared (mIR) have arisen as strong candidates for the task. Although a body of work exists that reports on their individual performances, a faithful comparison has not been established yet. We evaluated the performance of ^1^H-NMR, Attenuated Total Reflectance mIR (ATR-mIR), and NIR applied to fraud detection in Colombian coffee. For each technique, we built classification models for discrimination by species (*C. arabica* versus* C. canephora* (or* robusta*)) and by origin (Colombia versus other* C. arabica*) using a common set of coffee samples. All techniques successfully discriminated samples by species, as expected. Regarding origin determination, ATR-mIR and ^1^H-NMR showed comparable capacity to discriminate Colombian coffee samples, while NIR fell short by comparison. In conclusion, ATR-mIR, a less common technique in the field of coffee adulteration and fraud detection, emerges as a strong candidate, faster and with lower cost compared to ^1^H-NMR and more discriminating compared to NIR.

## 1. Introduction

Coffee is one of the most popular beverages in the world and an important trade commodity for producing countries. Out of the two most common species grown,* C. robusta *and* C. arabica*, the latter is preferred due to its characteristic taste, being less bitter than* C. robusta*. Colombia has been producing and exporting* C. arabica* coffee beans of the highest quality for at least one century, eventually garnering worldwide recognition.

In 2007* Colombian coffee* became a protected geographical indication, in recognition for its quality and thanks to decades of efforts from more than half a million coffee growers. Along with the increasing attractiveness of coffees from specific origins in international markets, this recognition represents a big economical* plus-value *for the label* 100% Colombian coffee.* During the same period, the overall production of Colombian coffee stagnated, forcing the country to import coffee beans from neighboring countries to supply its internal demand. This context raises the likelihood of fraud and thus creates a demand for robust methods to track the origin and ensure the quality of coffee beans. Cost-effectiveness is also a concern, since screening should preferably take place just before shipment and immediately after arrival, which means working at harbour installations and under limited operational costs.

Many efforts have been directed towards this aim. Techniques such as Gas Chromatography [1, 2], Gas Chromatography-Mass Spectrometry [3, 4], Liquid Chromatography [5], Inductively Coupled Plasma-Atomic Emission Spectroscopy [6], Isotope-Ratio Mass Spectrometry [7–13], Inductively Coupled Plasma-Mass Spectrometry [8, 11, 12], Mass Spectrometry [14], mid-infrared (mIR) spectroscopy [15–19], near infrared (NIR) spectroscopy [19–23], and Nuclear Magnetic Resonance (NMR) [24–27] have all been suggested to determine species or origin of coffee and thus detect adulteration with beans of lower quality. Moreover, combining output from several of these techniques in a single multivariate analysis has been shown to be useful for discriminating coffees from different continents [28].

Nontargeted approaches like fingerprinting based on NMR [25, 27], mIR [17–19], and NIR [19, 20, 23] spectroscopies have been reported to be capable of discriminating samples from nearby geographic origins. This is considered a harder task when compared with discrimination between transcontinental samples and an absolute must for fraud detection in the Colombian context, since fraud is more likely to involve coffee from neighboring countries.

One shortcoming of the existing literature on determination of origin in coffee is the lack of comparative studies that benchmark the performances of different analytical techniques. A remarkable exception is a recent article by Bona et al. [19] that compares the performance of mIR and NIR for origin determination and reports better results with the latter. Another contribution was found that reports on the comparison of both NIR and Attenuated Total Reflectance mIR (ATR-mIR) for the discrimination of coffee by species [16]. Benchmark comparative studies are particularly relevant when some of the techniques under consideration present major logistical advantages (sample preparation, analysis time, and cost per analysis), as is the case of IR spectroscopies compared to NMR. We thus present a comparative study of ^1^H-NMR, ATR-mIR, and NIR spectroscopies regarding their ability to discriminate* C. arabica* from* C. robusta* as well as Colombian coffees from other samples collected on nearby countries.

To guarantee a fair comparison, it is absolutely essential to ensure that all spectra used in the study were acquired on the same set of coffee samples. It is less obvious, however, whether one should utilize the same data analysis protocol for the three spectroscopic techniques or not. After careful consideration we decided that the answer to this question is negative: many different approaches for signal preprocessing (first derivative, second derivative, Multiplicative Scatter Correction (MSC), integral normalization, and probabilistic quotient normalization), variable standardization (mean-centering, unit variance scaling, Pareto scaling, and vast scaling), and Multivariate Discriminant Analysis (Projection on Latent Structures-Discriminant Analysis (PLS-DA), orthogonal PLS-DA (oPLS-DA), and orthogonal signal correction/PLS-DA) have been used in conjunction with NMR and/or infrared spectroscopy, and no unified standard exists that works the best with all kinds of spectroscopic data. Furthermore, since the computational cost of data analysis is currently not a concern in the field of coffee fraud detection, in practical scenarios one would use whatever chemometric approach yields the best results. For these reasons, we evaluated pipelines of different combinations of the methods just mentioned and report on the best results achieved for each type of spectroscopy.

## 2. Materials and Methods

### 2.1. Samples Collection and Preparation

A total of 97 samples of roasted coffee beans (7 minutes at 200°C) were collected from 14 countries worldwide during the years 2012 and 2013. These samples were provided by Almacafé S.A. (Colombia) and include 75 samples of* C. arabica* (from now on* Arabica*) coming from Colombia and nearby countries (Colombia: 34 samples, Guatemala: 15, Peru: 11, Brazil: 9, Costa Rica: 5, and Panama: 1) and 22 samples of* C. robusta* (from now on* Robusta*) coming from transoceanic countries (Vietnam: 8, India: 4, Uganda: 3, Indonesia: 3, Togo: 1, Tanzania: 1, Ivory Coast: 1, and Cameroon: 1). The* Arabic*a samples were selected from Latin American countries in order to reproduce the most likely real-life scenario for fraud detection in Colombian coffee.* Robusta* samples were introduced to verify the correctness of the chemometrics, as discussed at the beginning of Section 3. Samples were analyzed as they were shipped to our laboratory. There was no relationship between shipping time and coffee origin. The complete data set used in this work is available on Github (https://github.com/jwist/coffee-profiler/) and as Supplementary Materials (see Supplementary Materials available online at https://doi.org/10.1155/2017/7210463).

Both ATR-mIR and NIR spectra were acquired directly on the finely powdered samples (particle size 0.075 mm) provided by Almacafé S.A. Samples for ^1^H-NMR were prepared as follows: 200 mg of finely ground coffee powder was extracted with 1 mL chromatographic grade methanol at room temperature, followed by two minutes of agitation with vortex and 10 minutes of centrifugation, at 17°C. 450 *μ*L of the extract was transferred to the NMR tube, where 90 *μ*L of deuterated methanol with tetramethylsilane was added.

### 2.2. Analytical Techniques

ATR technology was used for the acquisition of mIR spectra to avoid pellet preparation, thus improving efficiency and reproducibility [29]. Spectra were obtained with a Nicolet™ iS™5 FTIR-ATR spectrometer operating in transmittance mode in the 650–4000 cm^−1^ region, with 64 scans per sample at intervals of 0.96 cm^−1^. The resulting spectra were stored as vectors of 3475 points after smoothing (Savitzky-Golay with a third-degree polynomial and a window size of 10 points).

NIR spectra were recorded from 4000 to 10000 cm^−1^ in reflectance mode, at intervals of 4 cm^−1^ and with 64 scans per sample, using a NIRFlex N-500 spectrometer (Büchi, Switzerland). Spectra were stored as 1500-point vectors, after smoothing with the Savitzky-Golay filter.

NMR experiments were performed in fully automatic mode on a Bruker Avance II 400 MHz spectrometer at 300 K. Accurate control of the sample temperature was achieved using BVT-1000 and BCU-1 units. After the frequency of the presaturation pulse (25 Hz) was accurately determined using a simple presaturation-excitation experiment (zgpr), 1D spectra (noesygpps) were recorded with a receiver gain of 90.5 and mixing time of 10 ms. After 4 dummy scans, 64 FIDs were added and stored in a vector of 131072 complex points. A 0.3 Hz exponential apodization function was applied prior to Fourier transformation; then each spectrum was phase corrected (zero-order only) and stored in a vector of 131072 real points using the Topspin software (V2.5 PL 6, Bruker Biospin A.G., Rheinstetten, Germany).

### 2.3. Chemometrics

#### 2.3.1. Preprocessing

Spectral regions containing meaningful signals were selected. For ATR-mIR wavelength range between 800 and 1800 cm^−1^ (1037 points) was selected, since it covers the fingerprint region of coffee samples and also contains the bands from carbonyl groups related to lipids and to molecules such as aliphatic and aromatic acids, vinyl esters, esters, aldehydes, ketones, lactones, and others, which confer different aroma to coffee [17, 30]. For NIR the range from 4000 to 7600 cm^−1^ (900 points) was selected, since the absorption bands related to caffeine, chlorogenic acid, water content, lipids, and aroma components among others are found in this range [30]. For NMR, first the signals of tetramethylsilane (−0.2–0.2 ppm) and methanol (3.14–3.55 ppm) were removed; then the spectrum was trimmed to the region containing visible peaks, from 0.5 to 9.5 ppm (80501 points), and binned to obtain a final vector of 1100 real points.

Second derivatives were computed for NIR and mIR spectra. In the case of NIR, an orthogonal signal correction (OSC, see [31]) filter was applied once. ATR-mIR spectra were normalized to a total integral of 100, while NIR spectra were normalized using MSC. None of these normalization steps were applied to NMR spectra. Last, spectroscopic variables were scaled by their standard deviations (unit variance scaling a.k.a. autoscaling).

Principal component analysis (PCA) was used to check data quality, to identify possible outliers or detect unexpected aggregation. For each coffee species and spectroscopic technique, samples outside 95% of Hotelling's *T* squared distribution on the first two principal components were rejected. Based on this test, 2 samples were rejected due to mIR, 5 due to NIR, and 5 due to NMR. In the case of NMR, most rejections were attributable to poor shims. Other rejections were most certainly a consequence of acquisition errors. Having removed the outliers, 66* Arabica *samples (Colombia: 30 samples, Guatemala: 14, Peru: 9, Brazil: 8, and Costa Rica: 5) and 19* Robusta* samples (Vietnam: 5, India: 4, Uganda: 3, Indonesia: 3, Togo: 1, Tanzania: 1, Ivory Coast: 1, and Cameroon: 1) remained.

All the preprocessing operations were performed on R [32] using in-house scripts and packages* stats* [32],* ChemoSpec* [33], and* rLims* [34].

#### 2.3.2. Classifiers

PLS modelling [35] was used to build the classifiers. The best models were obtained with oPLS-DA [35] for ATR-mIR, oPLS-DA for NMR, and PLS-DA for NIR. Since PLS methods are supervised and can thus be overtrained, it is mandatory to use a robust cross-validation scheme. All models were cross-validated through a standard 7-fold cross-validation [36]. We used a sampling algorithm that maintains the class ratio of the original set when selecting samples for training; this was done to avoid bias in the training/testing set due to one class being overrepresented. Furthermore, for the species classifier only 19 randomly selected* Arabica* samples were used to ensure a balanced representation of both species.

The performance of each technique was assessed on four bases:

(i) The mean and deviation of *Q*^2^ values over all models in the validation: here *Q*^2^ was defined as in [36]:
  (1) $$\begin{array}{c} Q^{2}=1-\frac{\sum_{i}^{}{\left(y_{i}-\hat{y_{i}}\right)}^{2}}{\sum_{i}^{}{\left(y_{i}-\underline{y}\right)}^{2}}, \end{array}$$
  where *y*_*i*_ is the observed value of the response variable for the *i*th sample, $\hat{y_{i}}$ is the corresponding predicted value, $\underline{y}$ is the mean, and the sums run over all samples in the testing set.
(ii) The Receiving Operator Characteristic (ROC) curve over all predictions: the predicted values from all models in the validation were stored in a single vector, the same was done with the observed values, and the ROC curve was computed using these vectors as input.
(iii) The distribution of areas under ROC curve (AUC): a ROC curve was computed for each model in the validation and the corresponding distribution of AUC values was visualized as a box plot.
(iv) The stability of the first loading: for each validation we computed the relative error (standard deviation/mean) of the first loading over the different models.

All the analyses were performed using* in-house* scripts written for the R software [32] and based on the pROC [37] and rLims [34] packages. All source code used is available on Github (https://github.com/jwist/coffee-profiler/) and as Supplementary Materials.

## 3. Results and Discussion

Two different classifiers were built for each spectroscopic technique: one for classification of beans by species (*Arabica* versus* Robusta*) and a second for classification of* Arabica* samples by origin (Colombia versus others). The former was a reference task used to check the adequacy of the chemometrics: according to the literature, all three spectroscopic techniques should be able to perfectly discriminate the samples by species [15, 16, 21, 22, 26, 27].

**Figure 1:**
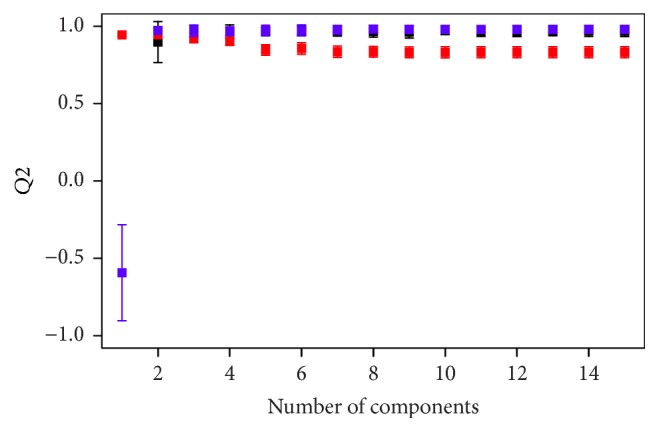
Results of discrimination by species, 7-fold cross-validation. Black represents ATR-mIR, red NIR, and blue ^1^H-NMR. The squares show the mean of the *Q*^2^ values averaged over all 7 models; the error bars show the standard deviation of these *Q*^2^. All techniques produced 100% accurate predictions at the top of the curves (2–4 components).

**Figure 2:**
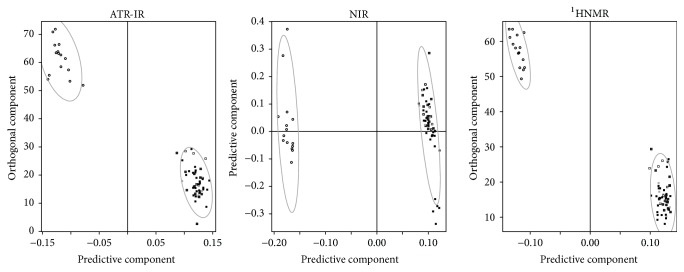
Scores plots of typical models for discrimination by species. These models were chosen from the top of the curves in Figure 1. By “typical” we mean that all models generated in the 7-fold validation presented pretty much the same behavior as the ones depicted. Squares:* Arabica* and circles:* Robusta*.

The results of discrimination by species are summarized in Figure 1. The plot shows the behavior of the *Q*^2^ values as we add predictive components (or subtract orthogonal components, in the case of oPLS) to the classifier. The error bars represent the range of variation of the *Q*^2^ values across the validation for the corresponding number of components. As more components are added to the classifier the *Q*^2^ values increase, showing the classifier's improvement. At some point, however, the *Q*^2^ values start to decrease. This means that the model is being overfit, causing predictions on the test samples to fail. The first optimum of each curve is then a key point for evaluating the performance of the corresponding spectroscopic technique. We found that all techniques presented comparable and very high *Q*^2^ values at their optima (around 2–4 components) with very low variation across the validation, a clear sign of top performance. In fact, they were all able to successfully predict the species of all test samples. This was the expected result, in agreement with what has been reported in previous publications, as said above. Since the three spectroscopic techniques presented 100% accuracy, the shapes of the corresponding ROC curves and AUC box plots are straightforward and we omitted them in Figure 1. Scores plots of typical models are presented in Figure 2, where the quality of the discrimination can be verified once more.

**Figure 3:**
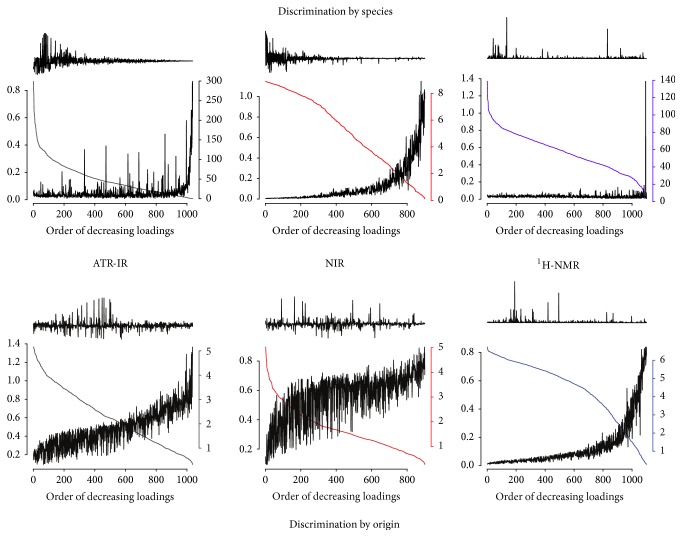
Loadings stability analysis. The first loadings vector was sorted before plotting. The smooth and coloured curve that drops from left to right corresponds to the values of the loadings in decreasing order. The oscillating curve falling from right to left corresponds to the relative error (std. dev./mean) of these loadings. Above each plot, the corresponding preprocessed spectrum prior to scaling, sorted to match the loadings' order. Results shown were obtained for the 7-fold validation, and results for the Monte Carlo validation are virtually identical.

Furthermore, in Figure 3 (top) we check the stability of the first loadings vector across the validation. This is an important factor in order to ensure that class-discriminating variables are independent of the selection of training data. In chemical terms this means that classes are distinguished by concentrations of a definite set of components. On the other hand, small loadings close to noise level are irrelevant for the discrimination but may vary wildly; to avoid this distraction, we sorted the loadings vector prior to plotting. It can be seen that high-valued loadings representing discriminating variables are very stable, oscillating in a <10% interval around the mean as we switch the training samples. This confirms that the discriminant variables do not depend on the sampling of the test set, meaning that the classification is indeed the result of variations in chemical composition depending on the species. Among the discriminating variables we found signals at 5.8, 6.2, 6.3, and 7.3 ppm attributed to Kahweol, a molecule that is mostly present in the* Arabica* species [38].

**Figure 4:**
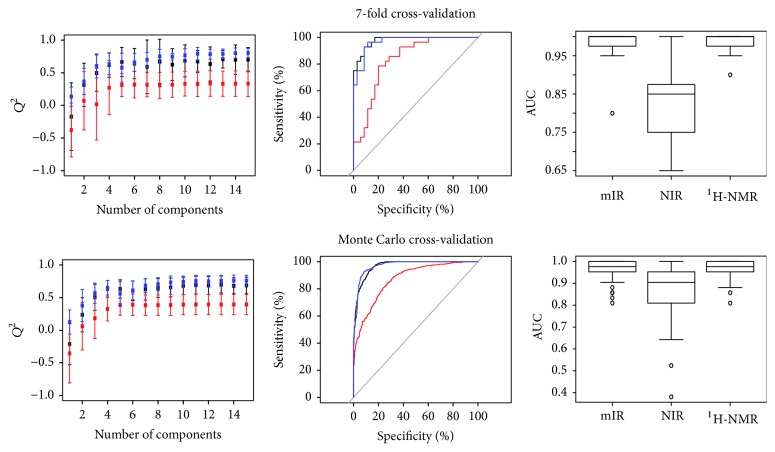
Results of discrimination by origin. In the coloured plots, blue is ^1^H-NMR, black is ATR-mIR, and red is NIR. *Q*^2^ values as a function of the number of components in the model (left), ROC curves (center), and area under the ROC curve for all models represented as box plots (right). The upper panel shows results using a standard 7-fold cross-validation, while the lower panel shows results obtained with a 100-model Monte Carlo cross-validation.

**Figure 5:**
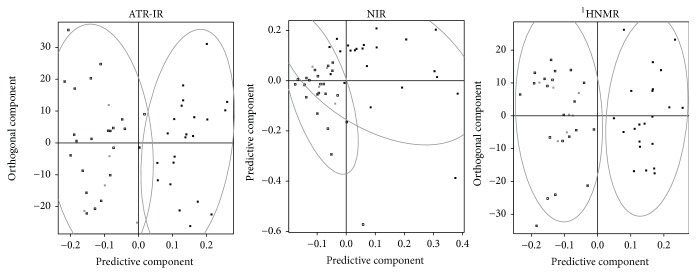
Scores plots of typical models for discrimination by origin. These models were chosen from the top of the curves in Figure 4 (left). By “typical” we mean that their *Q*^2^ values are the most frequent over the 100-model sample generated in the Monte Carlo validation. Black squares: Colombia and white squares: other countries.

Having checked the adequacy of our chemometrics, we proceeded to compare the spectroscopic techniques regarding their potential for fraud detection in Colombian coffee.  Figure 4 presents the results of this comparison regarding discriminating power. No technique reached a 100% accuracy, so this time we analyzed the ROC curves and AUC on top of the Q^2^ values. Scores plots of typical models are shown in Figure 5.

Figure 4 (top) summarizes the results obtained using 7-fold cross-validation. It is clear that NIR performed the worst, with the lowest *Q*^2^ values (left) and worst rate of accurate predictions as revealed by the ROC curves (mid) and AUCs (right). ^1^H-NMR and ATR-mIR, on the other hand, appear to yield equally high-quality results. However, we note that the ROC curves present “low resolution” due to the limited sampling achieved in a cross-validation that uses only 7 models. Suspecting that the inability to rank ^1^H-NMR and mIR could be due to this factor, we ran a Monte Carlo cross-validation with a sampling size of 100 models (Figure 4, bottom). The larger number of models computed this way improved the resolution of the ROC curves (mid) but the tie between ^1^H-NMR and ATR-mIR remained.

Interestingly, these results are contrary to those found by Bona et al. [19]. In this recent publication, which was probably the first to compare results of two spectroscopic techniques applied to the determination of origin in coffee, better results were reported for NIR than for mIR using KBr pellets. On the contrary, we found that NIR fell short of the desired discriminating power, while ATR-mIR matched the performance of ^1^H-NMR. The only two evident differences between both studies were that we used ATR technology to acquire the mIR spectra and that they were discriminating coffees within the same country (Brazil). Different biomarkers may be responsible for the differences between within-country samples and between off-country samples, which could potentially explain why we found opposing results. Regarding the use of ATR technology, previous applications of IR spectroscopy in food science have reported superior results using ATR-mIR for vitamin C analysis [39] and for determination of milk fatty acid content [40] when compared to other IR spectroscopy techniques. At the same time, Pillonel et al. [41] report better results for NIR when compared to ATR-mIR for determination of origin in cheese and achieved even better discrimination with mIR transmission spectroscopy (mIR/Tr). Then, it must be noted that sample preparation protocols differed, with ATR-mIR spectra being acquired on unprocessed slices, while NIR and mIR/Tr were taken on grated and further processed cheese. These differences may very well account for the quality of the results to a greater degree than the different spectroscopic techniques applied.

## 4. Conclusions

We provide what, to the best of our knowledge, the first benchmark is of Nuclear Magnetic Resonance (NMR) and state-of-the-art infrared spectroscopic techniques (near infrared (NIR) and Attenuated Reflectance mid-infrared (ATR-mIR)) for the origin determination of coffee. NIR spectroscopy, while arguably being the most attractive technique in terms of efficiency and cost, was found to yield results that put its ability to successfully classify Colombian coffee samples in doubt. Previous results by the authors were confirmed regarding the satisfying performance of ^1^H-NMR. On the other hand, ATR-mIR emerged as an attractive experimental setup due to its competitive performance, simpler implementation, and shorter time of analysis. We have thus provided valuable insight into the potential for fraud detection of three important spectroscopic techniques.

Our results regarding the relative performances of mIR and NIR were opposite to those of Bona et al. [19]. However, this disagreement is not inexplicable in light of the differences between these studies and considering previous findings on the application of IR spectroscopy to food science. Further research and comparative studies are required to understand the root of the disagreement, thus taking another step towards turning efficient coffee fraud detection by nontargeted chemical analysis in a practical reality.

## Supplementary Material

The supplementary material contains the original spectra and the necessary R scripts, as a zip file, to reproduce the results presented in this manuscript.
